# Supplementary material for: Cross-platform social dynamics: an analysis of ChatGPT and COVID-19 vaccine conversations
Source: Sci Rep. 2024 Feb 2;14:2789. doi: 10.1038/s41598-024-53124-x (PMC10837143; doi:10.1038/s41598-024-53124-x)
Supplement: Supplementary file 1 — Supplementary Information. [file 41598_2024_53124_MOESM1_ESM.pdf]

Supporting Information

COVID-19 Vaccination Dataset

In this section, we present an overview of the characteristics of datasets collected across different platforms related to COVID-19 vaccination debates. Keywords that we employed to collect data is as follows: *no-vax, novax, novaxx, anti-vax, pro-vax, dose, doses, dosers, dosed, pharmaceutical, pharmaceuticals, pharmacies, pharmacist, pharmacists, pharmacology, pharmacotherapy, pharmacy, pharming, pharmings, vaccina, vaccinal, vaccinas, vaccinate, vaccinated, vaccinates, vaccinating, vaccination, vaccinations, vaccinator, vaccinators, vaccine, vaccinee, vaccinees, vaccines, vaccinia, vaccinial, vaccinias, immune, immunes, immunise, immunised, immunises, immunising, immunities, immunity, immunization, immunizations, immunize, immunized, immunizer, immunizers, immunizes, immunizing, immunoassay, immunoassayable, immunoassays, immunoblot, immunoblots, immunoblotting, immunoblottings, immunochemical, immunochemist, immunochemistry, immunochemists, immunocompetent, immunodeficient, immunodiagnoses, immunodiagnosis, immunodiffusion, immunogen, immunogeneses, immunogenesis, immunogenetic, immunogenetics, immunogenic, immunogenicity, immunogens, immunoglobulin, immunoglobulins, immunologic, immunological, immunologically, immunologies, immunologist, immunologists, immunology, immunomodulator, immunopathology, immunoreactive, immunosorbent, immunosorbents, immunosuppress, immunotherapies, immunotherapy*.

Figure 4.a shows the cumulative count of content related to vaccine debates over the span of three months. Figure 4.b presents the distribution of interactions per platform. Interactions are a measure of user engagement with posts on social media platforms, and the definition can vary depending on the platform. On Twitter, interactions are the sum of likes, quotes, retweets, and replies for each post. On Instagram and YouTube, interactions are composed of likes and comments.

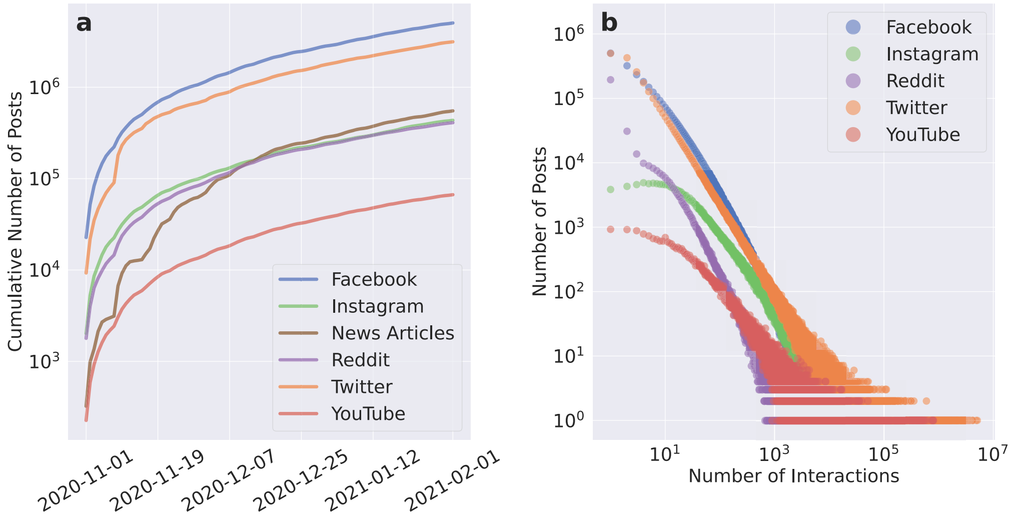

Figure 4. Cumulative number of unique posts about COVID vaccine discussion across various platforms (a) and distribution of interaction volume versus number of posts on different platforms (b).

Facebook Spam Content

Upon analyzing data related to ChatGPT discussions on Facebook, we observed some sharp strange dents in the cumulative number of posts and cumulative number of users. We then checked the dataset and found out there are many posts with similar styles publishing explicit or totally unrelated content in mass numbers that was inevitable to ignore. Our investigation resulted in flagging posts that used either of the following strings in their content: "#reels #chatgpt", "Video Funny Amazing #fyp #viral", and "#reeel #cr7 #chatgpt". We then proceed with plotting these flagged posts alongside the normal ones as shown in Figure 5. We observed orders of magnitude differences in the number of published posts per day and received interactions for these spam content, indicating that our efforts in removing these spam content were successful.

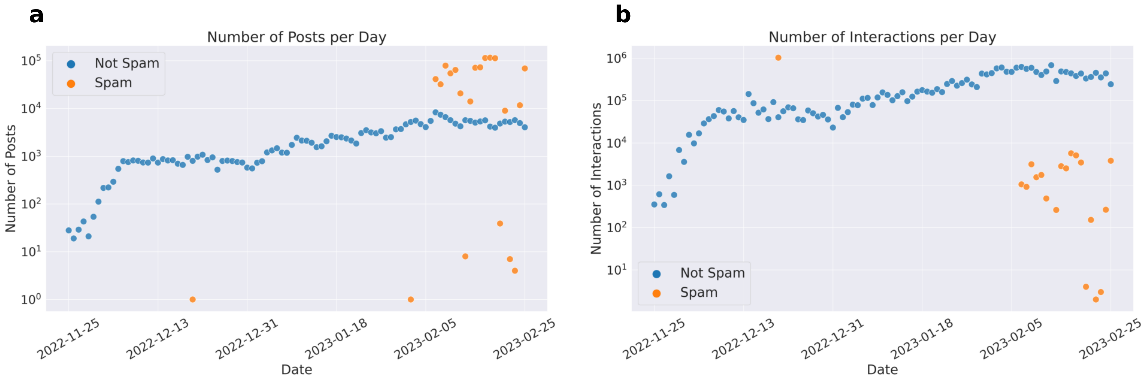

Figure 5. Number of daily posts and interactions for discussions about ChatGPT on Facebook. Content that we flagged as spam shows a totally different behavior.
